# Behavioral and Psychosocial Correlates of Gender Differences in Adolescent Mental Health: A Regional Cross-Sectional Study in Northern Italy

**DOI:** 10.3390/bs16050812

**Published:** 2026-05-19

**Authors:** Christian J. Wiedermann, Verena Barbieri, Giuliano Piccoliori, Doris Hager von Strobele Prainsack

**Affiliations:** Institute of General Practice and Public Health, Claudiana College of Health Professions, 39100 Bolzano, BZ, Italy

**Keywords:** adolescents, mental health, gender differences, depressive symptoms, anxiety, school stress, problematic internet use, sleep, South Tyrol, COP-S

## Abstract

Background: Gender differences in adolescent mental health are well documented; however, the extent to which modifiable behavioral and psychosocial factors account for the excess of mental health problems in females remains insufficiently quantified. Methods: Data from the 2025 Corona and Psyche South Tyrol (COP-S) survey comprised a base sample of 2428 adolescents aged 11–19 years (51.4% males) with valid self-reported data. Multivariable regression analyses were conducted on 1448–1603 adolescents (depending on the outcome) who provided complete responses to the relevant predictor and outcome measures. Gender differences in depressive symptom scores (PHQ-2), generalized anxiety symptom scores (SCARED-GAD), and emotional/behavioral difficulties (SDQ) were examined using Mann–Whitney U and chi-square tests. Multivariable linear regression models were used to assess the associations between mental health outcomes and the ten predictors. Gender effects were quantified by comparing standardized regression coefficients from unadjusted and adjusted models. Results: Female adolescents reported higher generalized anxiety symptoms (median 6 vs. 4; rank-biserial r = 0.24), depressive symptoms (r = 0.13), and emotional/behavioral (r = 0.08) scores than male adolescents. School stress, problematic Internet use, and sleep-onset difficulties were the factors most strongly associated with all three outcomes (all *p* < 0.001). After multivariable adjustment, gender remained significantly associated with generalized anxiety symptoms (β = 0.18) and depressive scores (β = 0.09) but no longer reached significance for emotional/behavioral scores (β = 0.04, *p* = 0.078). The attenuation of the gender effect ranged from 25.3% for generalized anxiety symptoms to 37.1% for depressive symptoms and 58.5% for emotional/behavioral difficulties. Conclusions: Gender differences in adolescent mental health were substantially attenuated after adjustment for modifiable behavioral and psychosocial factors, with the gender difference in emotional/behavioral scores no longer statistically significant after adjustment. Persistent gender disparities in generalized anxiety symptoms suggest that mechanisms beyond the measured behavioral correlates may contribute to this gender difference and warrant further investigation.

## 1. Introduction

Mental disorders are the leading cause of disability among children and adolescents globally. Meta-analyses estimate a 13.4% prevalence among this group (Polanczyk et al., 2015), supported by Global Burden of Disease data, which show an 11.6% prevalence for ages 5–24 (Kieling et al., 2024). Subthreshold psychological distress is more common and affects academic, social, and well-being aspects. Adolescent mental health issues often persist into adulthood, impacting education, work, health, and social outcomes (Kessler et al., 2005). Understanding the factors associated with adolescent mental health is crucial for public health research and preventive interventions.

Gender differences in adolescent mental health have been documented. Girls report more depressive symptoms, anxiety symptoms, and psychosomatic complaints than boys, with disparities increasing from mid-adolescence. These differences appear during development, with internalizing symptoms diverging in early to mid-adolescence (Barch et al., 2021; Wade et al., 2002). Longitudinal data show that internalizing symptoms rise in girls but remain stable or decline in boys, with a female-to-male depressive symptom ratio of 2:1 to 3:1 by late adolescence (Breslau et al., 2017; Daly, 2022). Boys more often show externalizing difficulties, such as conduct disorder and hyperactivity/inattention, with male-to-female ratios of approximately 2:1 in community samples (Fairchild et al., 2019; Zahn-Waxler et al., 2008). In the most recent (2025) wave of the Corona-and-Psyche South Tyrol (COP-S) survey series, which has been conducted in 2021, 2022, 2023 and 2025 (Barbieri et al., 2022, 2024, 2026b, 2023b), 40% of female adolescents screened positive for at least one mental health problem compared with 27% of male adolescents, with female adolescents reporting higher generalized anxiety symptom rates throughout the series (Barbieri et al., 2026b).

Gender-differentiated prevalence patterns are not fully understood, but cognitive and psychosocial factors play a role in this. Meta-analyses indicate that adolescent girls ruminate more than boys, mediating stress and depressive symptoms (Michl et al., 2013; Rood et al., 2009). Girls experience more interpersonal stress and use emotion-focused coping, which is linked to psychological symptoms (Hampel & Petermann, 2006). Restrictive gender norms partly mediate cultural differences in depression (Koenig et al., 2021), while self-reliant traits protect against depression (Lin et al., 2021). These findings suggest that female internalizing psychopathology results from cognitive vulnerabilities, gendered stress, and sociocultural constraints, not a single cause.

Several modifiable behavioral factors contribute to gender differences in adolescent mental health, with sleep disturbance being a key factor. Sleep-onset difficulties are more strongly linked to depressive symptoms than sleep duration (O’Callaghan et al., 2021), and a shorter sleep duration raises mood problem risks by 55% (Short et al., 2020). Sleep disturbances predict later internalizing symptoms regardless of initial psychopathology (Scott et al., 2021). Girls report shorter weekday sleep and worse sleep quality than boys, with stronger links between sleep and depression in females (Goldstone et al., 2020; Lemke et al., 2023). Poorer sleep in girls may partly explain their higher depression and anxiety symptoms.

The use of digital media is relevant. Reviews show minimal links between ordinary social media use and youth depression and anxiety, with causation disputed (Odgers & Jensen, 2020). Problematic Internet use (PIU), marked by preoccupation, loss of control, and impairment, is moderately associated with depression, anxiety, and reduced well-being in adolescents (Cai et al., 2023). Gender differences are notable: girls engage more in social comparison and addictive networking, whereas boys have higher problematic gaming rates, affecting their mental health (Cai et al., 2023). Barbieri et al. found that PIU predicts mental health issues in both genders, with similar effect sizes (Barbieri et al., 2026b). These analyses did not explore whether the gender gap in mental health outcomes is attenuated after accounting for PIU and other factors.

Perceived social support is crucial for the mental health of adolescents. It predicts lower depression, anxiety, and suicidal ideation in young adulthood (Scardera et al., 2020). Family support and friendship quality in early adolescence predict fewer depressive symptoms in adolescents who have experienced prior adversity (van Harmelen et al., 2016). Peer relationship quality is strongly associated with depression risk, especially in girls (Letkiewicz et al., 2023). Social support reduces chronic stress and fosters adaptive coping, potentially buffering gender-differentiated stressors.

School-related stress is a key correlate of adolescent mental health, with links to depressive and anxiety symptoms (Högberg et al., 2020). Female adolescents report more school stress than males, suggesting that differential burden may affect gender disparities in internalizing symptoms. Physical activity protects adolescent mental health, with dose–response relationships for depression and anxiety (Ruiz-Ranz & Asín-Izquierdo, 2025). Boys engage in more physical activity than girls, possibly explaining the mental health difficulties in females. Health literacy, the capacity to access, understand, and apply health information, is linked to mental health outcomes in adolescents (Paakkari et al., 2020), although its role in gender differences is underexplored.

Despite extensive research on gender differences in adolescent mental health, there remains a critical gap. Most studies either examine predictors within each gender separately or document prevalence differences without addressing whether the gender gap is due to modifiable behavioral and psychosocial exposures. It is unclear to what extent factors such as sleep disturbance, digital media use, physical inactivity, school stress, perceived social support, and health literacy, which may vary across genders, contribute to the excess of mental health issues in females, and whether gender differences persist independently after adjusting for these factors. Most evidence comes from demographically limited samples, and studies focusing on a single predictor cannot assess the contributions of multiple factors or determine whether gender effects remain after joint adjustment. This question is relevant for intervention design: if gender differences in mental health are substantially attenuated after adjustment for modifiable factors, this would suggest that interventions addressing those factors could help reduce the mental health gap between male and female adolescents.

This study used data from a survey of adolescents aged 11–19 years in South Tyrol, Northern Italy, as part of the COP-S survey series (Barbieri et al., 2023a, 2024, 2026b). This study aims to: (1) examine gender differences in mental health indicators such as depressive and anxiety symptoms and related behavioral and psychosocial factors; (2) investigate multivariable associations between these factors and mental health outcomes, identifying the most relevant correlates; and (3) quantify the extent to which gender differences in mental health are statistically attenuated by behavioral and psychosocial factors, comparing unadjusted and adjusted gender effects. This analysis, which has not been previously performed on this dataset, addresses whether the excess female adolescent mental health issues reflect gender effects or modifiable exposures that differ between genders.

## 2. Methods

### 2.1. Study Design and Setting

This cross-sectional study analyzed data from the fourth wave of the COP-S survey series, which was conducted between 17 March and 13 April 2025. The COP-S series is a repeated cross-sectional, large school-based regional survey of children and adolescents in South Tyrol, a bilingual autonomous province in Northern Italy bordering Austria with approximately 530,000 inhabitants. Previous waves were conducted in 2021, 2022, and 2023 (Barbieri et al., 2023a, 2024). Each wave recruited an independent sample, and participants were not followed across waves. The primary objective of the survey series was to monitor post-pandemic mental health trends among the youth population of South Tyrol and examine the associated behavioral, psychosocial, and sociodemographic factors. The survey was administered via the SoSci Survey platform (Version 3.2.46; SoSci Survey GmbH, Munich, Germany) and was available in German and Italian versions.

### 2.2. Participants and Recruitment

The questionnaire was distributed to families of schoolchildren across South Tyrol via personalized email links sent through school directorates to approximately 40,000 families in South Tyrol. A reminder was sent to all families two weeks after the initial invitation. Both parental proxy and adolescent self-report versions were included. Adolescents could complete the self-report form independently after their parents completed the proxy version; due to the anonymous online format, independent completion could not be verified.

To assess representativeness, the sociodemographic profile of the analytic sample was compared with the resident adolescent population of South Tyrol (11–19 years; *n* = 51,846 as of 31 December 2024) using publicly available data from the Provincial Statistics Institute (ASTAT). The gender distribution of the analytic sample (48.6% female) closely matched the regional reference population (48.0% female), while younger adolescents aged 11–14 years were modestly overrepresented (48.1% vs. 44.0%). The analytic sample corresponded to approximately 4.7% of all South Tyrolean adolescents in this age range (Supplementary Table S2).

The present analysis focused exclusively on adolescent self-reported data from participants aged 11–19 years. Adolescents were included if they confirmed their participation in the self-report questionnaire and attended lower secondary school, upper secondary school, a vocational track, or another formal educational setting. Adolescents who reported attendance at primary school (*n* = 122) were excluded to maintain consistency with the target age range, as were participants with missing school-type information. All remaining adolescents were retained as the base sample for descriptive analyses (*n* = 2428), irrespective of the completeness of the behavioral and mental health modules. The present sample definition differs from that applied by Barbieri et al. (2026b) in their gender-specific analysis of the same COP-S wave, which was restricted to adolescents with complete data on all three mental health instruments (*n* = 1471). The broader inclusion criteria adopted in this study were chosen to retain the full range of sociodemographic variability and to enable the use of continuous mental health scores as outcomes. Regression models accommodate item-specific missingness through listwise deletion per model, resulting in analytic samples of approximately 1400–1600 adolescents for each outcome. A comparison of complete versus sparsely completed self-report subgroups drawn from the same COP-S 2025 wave is reported in Supplementary Material 1 of Barbieri et al. (2026b), showing no substantive differences in demographic composition but modest differences in behavioral factors, such as elevated problematic Internet use, perceived school burden, and low social support.

Data collection was not in classrooms; invitation links were emailed for home completion. The SoSci Survey ensured anonymity, storing no names, birth dates, IPs, or emails, and no code linked adolescent and parental records. School directorates sent invitations without accessing responses. Adolescents completed their questionnaires independently, assured their answers were private. The study’s purpose, voluntary participation, and data anonymity were explained, with informed consent obtained. The anonymous, self-administered format aimed to reduce bias, though it cannot be fully excluded in self-reports.

### 2.3. Measures

#### 2.3.1. Mental Health Outcomes

Three validated screening instruments were used to assess self-reported mental health. All instruments were available in validated German and Italian versions and were applied consistently across all COP-S waves.

Depressive symptoms were assessed using the Patient Health Questionnaire-2 (PHQ-2), a two-item instrument asking about the frequency of depressed mood and anhedonia over the past two weeks, rated on a four-point Likert scale from 0 (nearly never) to 3 (nearly every day), yielding a total score ranging from 0 to 6 (Löwe et al., 2005). The PHQ-2 is recommended for use in adolescents aged 12 years and older and has been validated in the relevant cultural context (D’Argenio et al., 2013; Schuler et al., 2018). A total score of ≥3 was applied as the threshold for elevated depressive symptoms, consistent with established cut-off recommendations and prior COP-S publications (Barbieri et al., 2026b).

Anxiety symptoms were assessed using the Generalized Anxiety Disorder subscale (GAD-9) of the Screen for Child Anxiety-Related Emotional Disorders (SCARED). This nine-item subscale asks adolescents to rate the frequency of generalized anxiety symptom-related cognitions and behaviors on a three-point scale from 0 (not or hardly true) to 2 (very or often true), yielding a total score of 0–18 (Birmaher et al., 1999). German and Italian validation studies confirmed adequate psychometric properties in adolescent community samples (Crocetti et al., 2009; Weitkamp et al., 2010). A total score of ≥9 was used as the threshold for elevated generalized anxiety symptoms, consistent with prior COP-S applications. In the present study, this subscale is referred to as SCARED-GAD.

Emotional and behavioral difficulties were assessed using the Strengths and Difficulties Questionnaire (SDQ) (Goodman, 2001). The total difficulty score was computed from four subscales: emotional symptoms, conduct problems, hyperactivity/inattention, and peer relationship problems, each comprising five items rated on a three-point scale (0, 1, 2), yielding a total score of 0–40. Higher scores indicate greater difficulty. Scores were dichotomized according to established cut-offs, with borderline and abnormal range scores (≥17) classified as elevated, consistent with prior COP-S analyses (Barbieri et al., 2026b). The SDQ has been validated in German- and Italian-speaking populations (Goodman, 2001).

#### 2.3.2. Behavioral and Psychosocial Correlates

Physical activity was assessed by asking adolescents how many days in the past week they had engaged in at least 60 min of sports or physical activity, rated on an eight-point scale from 1 (0 days) to 8 (7 days). For the present analyses, the responses were treated as continuous variables representing days per week with sufficient physical activity.

School stress was assessed via adolescent self-report using a single item asking about the perceived burden of schoolwork, rated on a four-point Likert scale (1 = ‘not at all’, 2 = ‘somewhat’, 3 = ‘fairly strongly’, 4 = ‘very strongly’), consistent with items used in the Health Behavior in School-aged Children (HBSC) survey series and prior COP-S analyses (Barbieri et al., 2024). For the multivariable regression analyses, the item was entered as a metric covariate, treating the response scale as approximately equidistant, in line with the common practice for single-item stress measures in large-scale adolescent health surveys. This item has demonstrated adequate criterion validity in large-scale epidemiological surveys of adolescent health (Högberg et al., 2020).

Health literacy was assessed using the Health Literacy for School-Aged Children (HLSAC) scale from the HBSC study (Paakkari et al., 2020). This ten-item instrument asks adolescents to rate health-related competence statements on a four-point Likert scale from 1 (not at all true) to 4 (absolutely true), yielding total scores ranging from 10 to 40. Higher scores indicate better health literacy levels. The HLSAC demonstrated high internal consistency in the present sample (Cronbach’s α = 0.892) (Barbieri et al., 2026b).

Sleep was assessed using two items. Sleep-onset difficulties were operationalized from a single HBSC-derived item asking how often the adolescent had difficulty falling asleep during the past six months, with responses rated on a five-point scale (daily, more than once a week, about once a week, about once a month, rarely or never). Responses were dichotomized such that any frequency of at least once a week (i.e., all categories except “rarely or never”) was coded as sleep-onset difficulties, consistent with the HBSC scoring practice applied in previous COP-S waves (Barbieri et al., 2024). Late bedtime was defined as a self-reported habitual school day bedtime after 23:00 h, derived from a six-category response scale.

PIU was assessed using the Generalized Problematic Internet Use Scale 2 (GPIUS-2) (Caplan, 2010), comprising 15 items rated on an eight-point Likert scale from 1 (definitely disagree) to 8 (definitely agree), yielding a total score of 15–120. Higher scores indicate greater problematic Internet use. Validated German and Italian versions were used (Barke et al., 2014; Casale et al., 2014). In the present sample, Cronbach’s α was 0.925 (Barbieri et al., 2026b).

Social media use intensity was assessed using the Bergen Social Media Addiction Scale (BSMAS) (Andreassen et al., 2017), a six-item instrument rated on a five-point Likert scale from 1 (very rarely) to 5 (very often), yielding total scores ranging from 6 to 30. Higher scores indicate more intensive social media use.

Perceived social support was measured using the Multidimensional Scale of Perceived Social Support (MSPSS) (Zimet et al., 1988), a 12-item instrument assessing perceived support from family, friends, and significant others, rated on a seven-point Likert scale ranging from 1 (strongly disagree) to 7 (strongly agree). Three subscale scores were computed (range: 1–7). Due to collinearity between subscales (detailed in the Statistical Analysis section), an MSPSS total score was used in multivariable analyses. Cronbach’s α was 0.976 in the present sample (Barbieri et al., 2026b).

#### 2.3.3. Sociodemographic Variables

Gender was assessed using adolescent self-report, with response options of boy, girl, or diverse. For the present analyses, gender was operationalized as a binary variable (0 = male, 1 = female). Two adolescents who identified as diverse could not be retained as a separate group, as the group size was insufficient for meaningful statistical analysis; this constraint, and its conceptual implications, are addressed in the Limitations section. We acknowledge that a binary operationalization does not capture the full diversity of adolescent gender identity.

Age was reported in completed years by the adolescents. The analytic sample ranged from 11 to 19 years of age.

Parents reported school type, which was classified as a lower secondary school (grades 6–8), upper secondary school (grades 9–13), vocational school, or other. In South Tyrol, lower and upper secondary schools follow the standard Italian school system within the German- or Italian-language educational track, and vocational schools offer profession-oriented training programs of shorter duration (Barbieri et al., 2026a).

Parents reported the school language as German, Italian, or Ladin.

Parents reported their family language as German, Italian, Ladin, or other.

Migration background was defined as at least one parent born outside Italy and reported by parents as a binary variable.

Single-parent households were reported by parents as a binary variable.

Parental education was classified using the Comparative Analysis of Social Mobility in Industrial Nations (CASMIN) index into three ordinal categories: low (no or lower secondary education), medium (upper secondary or vocational training), and high (tertiary education) (Brauns et al., 2003).

Family affluence was assessed using the Family Affluence Scale III (FAS III) (Currie et al., 2024), a six-item parent-reported measure of material wealth indicators, including car ownership, computer access, own bedroom, bathroom, dishwasher, and vacation frequency. The total score ranged from 0 to 13, with higher scores indicating greater family affluence.

### 2.4. Statistical Analyses

Descriptive statistics were calculated for all study variables. Categorical variables are reported as frequencies and proportions, and continuous and ordinal variables are summarized as medians with minimum–maximum ranges and, where informative, means with standard deviations. Gender comparisons for continuous and ordinal variables were conducted using the Mann–Whitney U test, with the rank-biserial correlation coefficient r = |Z|/√N reported as the effect size. Categorical variables were compared using Pearson’s chi-square test, with Cramér’s V reported as the effect size (Cramér’s V equals the phi coefficient for 2 × 2 tables). Following Cohen (1988), effect sizes were interpreted as negligible (<0.10), small (0.10–0.29), moderate (0.30–0.49), or large (≥0.50).

Prior to multivariable modelling, bivariate associations among all study variables were examined using Spearman rank-order correlations (rs), appropriate given the ordinal nature of several predictors and the non-normal distribution of the outcome variables. The full correlation matrix is presented in Supplementary Table S1. Variable pairs with |rs| > 0.70 were considered potentially collinear. Based on this criterion, GPIUS-2 and BSMAS were found to be collinear (rs = 0.72); GPIUS-2 was retained as the primary digital media predictor, given its broader conceptual scope encompassing problematic Internet use. The MSPSS total score was used instead of the individual subscales to avoid collinearity among the three subscales (rs = 0.58–0.74).

Multivariable associations between behavioral and psychosocial predictors and each mental health outcome were examined using simultaneous entry ordinary least squares (OLS) linear regression analysis. Three separate models were estimated, one for each outcome (PHQ-2, SCARED-GAD, and SDQ total difficulties score). The following covariates were entered into each model: gender, age, physical activity, school stress (self-report), health literacy (HLSAC), sleep-onset difficulties, late bedtime, PIU (GPIUS-2), perceived social support (MSPSS total), and family affluence (FAS III). The behavioral and psychosocial variables included in the multivariable models are referred to throughout the manuscript as correlates or statistical predictors of the mental health outcomes, denoting their role within the regression framework, rather than as causal risk factors. Variance inflation factors (VIF) were used to confirm the absence of multicollinearity in the fitted models (threshold: VIF < 5). The three outcome scores showed the bounded, right-skewed distributions typical of symptom screening instruments in general-population samples, including a pronounced floor effect for the PHQ-2. Ordinary least squares (OLS) regression was retained as the primary analytical approach because of its direct interpretability and the large analytic sample, under which the coefficient estimates remain asymptotically valid despite non-normal residuals. To verify that the bounded and skewed nature of the outcomes did not distort the substantive conclusions, each model was re-estimated as a sensitivity analysis using a Poisson generalized linear model with a log link and robust (Huber–White sandwich) standard errors, which models the count-type outcome distribution directly and does not assume normality. A negative binomial specification was examined first, but the dispersion parameter for the PHQ-2 model was near zero, indicating that the Poisson model was appropriate; model fit was assessed using the deviance/df ratio, and robust standard errors were used throughout to provide valid inference in the presence of any overdispersion.

To examine whether the gender association varied across the broad age range of the sample, a gender × age interaction term (using mean-centered age) was added to each of the three regression models in a separate step, and the resulting change in model fit was evaluated.

Because school type differed by gender, each model was additionally re-estimated with school type (lower secondary, upper secondary, vocational, or other; lower secondary as reference) entered as a further covariate, to verify that this did not alter the gender association.

Missing data were handled according to the type of analysis. Descriptive statistics and the Spearman correlation matrix were based on all cases with valid data for each variable or variable pair, respectively. The multivariable regression models used listwise deletion, so that each model was estimated on the subset of adolescents with complete data on the outcome and all covariates entered in that model. As a sensitivity analysis addressing potential bias from this item-level missingness, participants in the base sample were classified according to whether they had complete data on all regression variables or were excluded by listwise deletion, and the two groups were compared on all regression variables using Mann–Whitney U tests and Pearson χ^2^ tests.

The attenuation of the gender effect following covariate adjustment was quantified as ((β unadjusted − β adjusted)/β unadjusted) × 100, following established change-in-estimate conventions (Greenland, 1989; MacKinnon, 2012). This measure represents the proportion of the unadjusted gender effect that is statistically attenuated after inclusion of the behavioral and psychosocial covariates. It should be interpreted descriptively, as statistical attenuation following covariate adjustment, and not as evidence of mediation or of a causal pathway; the cross-sectional design does not permit causal or mediational inference.

All analyses were conducted using the IBM SPSS Statistics software (version 25.0; IBM Corp., Armonk, NY, USA).

### 2.5. Use of Generative Artificial Intelligence

GenAI (ChatGPT, OpenAI) was used to assist in structuring and refining the manuscript text, including the formulation of the Introduction and Methods sections, based on the study protocol and validated instruments. Artificial intelligence (AI) was also used to synthesize and cross-reference the existing literature for clarity and contextualization. No generative AI was used for data collection, statistical analysis, or interpretation of the results. All content was reviewed and approved by the authors.

## 3. Results

### 3.1. Sample Characteristics

The analytic sample comprised 2428 adolescents aged 11–19 years, evenly distributed by gender (51.4% males). Age, family language, migration background, single-parent household, parental education, and urban residence were comparable between male and female adolescents (all V ≤ 0.04; see Table 1). School type and family affluence reached statistical significance in gender comparisons (*p* < 0.001 and *p* = 0.004, respectively); however, both effects were negligible in magnitude (V ≤ 0.09), reflecting the power of the large sample rather than substantive group differences.

### 3.2. Gender Differences in Mental Health Outcomes

Female adolescents scored higher than male adolescents on all three screening instruments, with the generalized anxiety symptoms domain showing the most pronounced gap. Elevated SCARED GAD-9 scores were nearly twice as prevalent in female as in male adolescents, corresponding to a small-to-moderate effect (V = 0.20; r = 0.24), the largest gender difference observed in the study. Depressive (PHQ-2) and general (SDQ) mental health scores also showed a significant female excess both at the cut-off and on the continuous scale, although with markedly smaller effects (V ≤ 0.08; r ≤ 0.13). Across all three domains, effect sizes remained below the conventional threshold for moderate effects, indicating that the observed gender disparities, while statistically robust, were of limited magnitude at the population level.

To clarify the composition of the gender difference in the SDQ total difficulties score, the four problem subscales and the prosocial subscale were compared between genders (Supplementary Table S5). The female excess in total difficulties was driven almost entirely by the emotional symptoms subscale, which showed the largest gender difference of all SDQ components (median 3.0 vs. 1.0; r = 0.24). The externalizing subscales showed no corresponding female excess: conduct problems did not differ significantly between genders (r = 0.04), and hyperactivity/inattention was slightly higher among male adolescents (r = 0.09). Peer relationship problems were marginally higher in female adolescents (r = 0.05), and female adolescents also reported somewhat higher prosocial behavior (r = 0.14). The comparatively small gender difference in the total difficulties score (r = 0.08) therefore reflects the combination of a substantial female excess in emotional symptoms with the absence of a female excess in the externalizing domains.

### 3.3. Gender Differences in Behavioral and Psychosocial Correlates

Behavioral and psychosocial data were available for 1448–1603 adolescents in each wave. Physical activity differed markedly by gender (V = 0.17), with male adolescents substantially more likely than female adolescents to report at least three active days per week; this was the second-largest effect observed after SCARED anxiety. Sleep problems and perceived burden from global crises were more frequent in female adolescents, although both effects were small (V ≤ 0.08). Late bedtimes on school days, problematic Internet use, perceived social support, and health literacy did not differ meaningfully between genders (all V ≤ 0.05, r ≤ 0.05), where nominal significance emerged on continuous scales, and the corresponding rank-biserial correlations were vanishingly small, again reflecting sample size rather than substantive group differences.

### 3.4. Multivariable Associations Between Correlates and Mental Health

The results of the three multivariable linear regression models are presented in Table 2. All models explained a substantial and comparable proportion of variance in mental health outcomes, with adjusted R^2^ values of 0.36 for depressive symptom scores, 0.35 for generalized anxiety symptom scores, and 0.42 for emotional and behavioral scores. The VIF ranged from 1.02 to 1.18 across all predictors and models, confirming the absence of multicollinearity in the dataset.

School stress was the most consistent and strongest correlate across all three outcomes. Higher self-reported school burden, entered as a metric predictor on the original four-point response scale, was associated with higher PHQ-2 (standardized β = 0.23), SCARED-GAD (β = 0.28), and SDQ total difficulties scores (β = 0.24; all *p* < 0.001). PIU (GPIUS-2) was also significantly associated with all three outcomes, with particularly strong associations with SDQ total difficulty scores (β = 0.38, *p* < 0.001) and comparable associations with depressive (β = 0.26) and generalized anxiety symptom scores (β = 0.24). Sleep-onset difficulties were independently associated with all three outcomes (β = 0.17–0.21; *p* < 0.001). Higher perceived social support (MSPSS total) was inversely associated with all outcomes (β = −0.07 to −0.16, all *p* ≤ 0.002), indicating a consistent protective role across mental health domains.

Gender showed a heterogeneous pattern across the three mental health domains after adjusting for behavioral and psychosocial factors. Female gender was significantly and independently associated with depressive scores (β = 0.09, *p* < 0.001) and generalized anxiety symptom scores (β = 0.18, *p* < 0.001), with the largest effect observed for generalized anxiety symptoms, where gender was the second strongest association after school stress. In contrast, the gender effect on SDQ total difficulties scores no longer reached statistical significance after covariate adjustment (β = 0.04, *p* = 0.078), indicating that the gender difference in emotional and behavioral scores was no longer statistically detectable after adjustment for the measured behavioral and psychosocial factors.

Several predictors showed outcome-specific associations. Late bedtime was independently associated with depressive symptom scores (β = 0.08, *p* = 0.002) but not with generalized anxiety symptom or SDQ scores. Physical activity was inversely associated with depressive symptom scores (β = −0.08, *p* < 0.001) and, more modestly, with generalized anxiety symptom scores (β = −0.05, *p* = 0.036), but was not a significant predictor of SDQ scores. Health literacy was significantly and inversely associated with SDQ scores (β = −0.10, *p* < 0.001) but not with depressive symptom or generalized anxiety symptom scores. Age was positively associated with depressive scores (β = 0.09, *p* < 0.001) and inversely associated with SDQ scores (β = −0.06, *p* = 0.011) but not significantly associated with generalized anxiety symptom scores. Family affluence (FAS-III) was not significantly associated with any outcome in the multivariate models, consistent with the near-zero bivariate correlations observed in Supplementary Table S1.

### 3.5. Gender Differences in Mental Health After Multivariable Adjustment

Table 3 presents the unadjusted and fully adjusted regression coefficients for gender across all three mental health outcomes. In unadjusted models, female gender was associated with significantly higher scores on all three outcomes (all *p* < 0.001), with the largest unadjusted effect observed for generalized anxiety symptoms (β = 0.24), followed by depressive symptoms (β = 0.14) and emotional and behavioral difficulties (β = 0.09).

After full adjustment for age, physical activity, school stress, health literacy, sleep behavior, problematic Internet use, perceived social support, and family affluence, the gender coefficient was substantially attenuated in all three models. The residual gender effect remained highly significant for generalized anxiety symptom (adjusted β = 0.18, *p* < 0.001) and depressive symptom scores (β = 0.09, *p* < 0.001) but no longer reached statistical significance for emotional/behavioral scores (β = 0.04, *p* = 0.078).

The magnitude of attenuation differed markedly among the outcomes. Adjustment for the full set of behavioral and psychosocial covariates reduced the gender effect on emotional/behavioral scores by 58.5%, on depressive scores by 37.1%, and on generalized anxiety symptom scores by 25.3%. The comparatively modest attenuation for generalized generalized anxiety symptoms indicates that the gender difference in generalized anxiety symptoms was less strongly attenuated by the measured behavioral and psychosocial factors than by the female excess in depressive and emotional/behavioral scores. Conversely, the near-complete attenuation of the gender effect on SDQ scores, such that it no longer reached statistical significance after adjustment, suggests that the gender gap in emotional and behavioral difficulties was largely attenuated by the measured covariates. The variables most likely contributing to this attenuation, given that they both differed significantly by gender in Table 1 and were significant predictors in Table 2, included physical activity, sleep-onset difficulties, and problematic Internet use.

Taken together, these findings indicate that gender differences in adolescent mental health persist independently of the behavioral and psychosocial factors examined, although a meaningful proportion of the gender effect, particularly for depressive symptoms and behavioral difficulties, was attenuated after adjustment for modifiable factors.

### 3.6. Sensitivity Analyses

To determine if item-level missing data in the regression models led to selection bias, participants with complete data on all regression variables (*n* = 1261) were compared to those excluded through listwise deletion (*n* = 1167) for each regression variable (Supplementary Table S3). Among the twelve variables examined, only age showed statistical significance, with a minimal effect size (median age of 15.0 years in both groups; r = 0.06). There were no significant differences between the groups in terms of behavioral, psychosocial, or mental health variables; specifically, problematic Internet use (*p* = 0.981), school stress (*p* = 0.115), and perceived social support (*p* = 0.265) did not differ meaningfully (all r ≤ 0.04). These findings suggest that listwise deletion did not significantly affect the selection of constructs included in the regression models.

A second sensitivity analysis examined whether the bounded, right-skewed distribution of the outcome scores affected the regression results. The three outcome scores were right-skewed, with skewness values of 1.54 (PHQ-2), 0.70 (SCARED-GAD), and 0.70 (SDQ total difficulties), and the proportion of participants at the scale floor was 42.9%, 9.6%, and 2.4%, respectively. Each model was re-estimated using a Poisson generalized linear model with robust standard errors (Supplementary Table S4). The deviance/df ratio indicated no overdispersion for the PHQ-2 model (1.05) and moderate overdispersion for the SCARED-GAD (2.74) and SDQ (2.26) models, the latter accommodated by the robust standard errors. The Poisson models yielded the same inferential conclusions as the main OLS models for all three outcomes: the gender association was statistically significant for depressive symptoms (incidence rate ratio [IRR] = 1.21, 95% CI [1.09, 1.34]) and generalized anxiety symptoms (IRR = 1.32, 95% CI [1.23, 1.43]), and non-significant for total difficulties (IRR = 1.04, 95% CI [0.98, 1.10]). The associations of the behavioral and psychosocial covariates were likewise consistent in direction and significance across the two modelling approaches. The main findings were therefore robust to the skewness, bounded range, and floor effects of the outcome scales.

A third sensitivity analysis tested whether the gender association varied across the age range studied. When a gender × age interaction term was added to each regression model, it was non-significant for all three outcomes—depressive symptoms (B = 0.03, *p* = 0.299), anxiety symptoms (B = −0.10, *p* = 0.319), and total difficulties (B = −0.09, *p* = 0.432)—and the gender main effect remained essentially unchanged in each model (standardized β changing by no more than 0.002). These results indicate that the gender differences in mental health symptoms were stable across early, middle, and late adolescence, and that the broad age range of the sample did not obscure age-specific variation in the gender effect.

## 4. Discussion

This regional cross-sectional study examined gender differences in adolescent mental health and associated behavioral and psychosocial factors among 2428 adolescents aged 11–19 years in South Tyrol, Italy. Three principal findings were obtained. First, female adolescents consistently showed higher symptom levels across all three mental health domains, with the largest and most clinically relevant disparity observed for generalized anxiety symptoms. Second, school stress and PIU were the factors most strongly and consistently associated with mental health outcomes across all three domains, followed by sleep-onset difficulties and perceived social support, with several additional predictors showing specific associations. Third, gender differences in mental health outcomes persisted after comprehensive multivariable adjustment, although a meaningful proportion of the gender effect was attenuated by the measured behavioral and psychosocial factors, most substantially for behavioral difficulties and depressive symptoms, and least for generalized anxiety symptoms.

### 4.1. Gender Differences in Mental Health Outcomes

The prevalence of elevated generalized anxiety symptoms scores in female adolescents (37.6%) was nearly double that of males (19.5%), with an effect size (r = 0.236) nearing moderate and much higher than that of depressive symptom scores (r = 0.134) and emotional/behavioral scores (r = 0.081). These results align with meta-analytic evidence showing a persistent female excess in internalizing psychopathology during adolescence (Madigan et al., 2023; Rutter et al., 2003) and replicate patterns from previous COP-S waves in 2021, 2022, and 2023, where females consistently had higher generalized anxiety symptom rates than males (phi = 0.176–0.189, all *p* < 0.001) (Barbieri et al., 2023a, 2024). Developmental models highlighting hormonal, cognitive, and social mechanisms that specifically increase anxiety in females support this pattern (Hale et al., 2010; McLaughlin & Nolen-Hoeksema, 2011).

The prevalence of elevated depressive symptom scores (PHQ-2 ≥ 3:13.8% female, 9.0% male) aligns with post-pandemic estimates from similar European settings (Madigan et al., 2023). Within the COP-S series, this 11.4% prevalence shows a decline from 15.4% in 2021 and 13.7% in 2022, with female rates dropping from 20.1% in 2021 to 13.5% in 2023 and stabilizing by 2025 (Barbieri et al., 2023a, 2024). The present sample included all adolescents with valid responses, unlike Barbieri et al., who restricted their study to complete cases (Barbieri et al., 2026b), causing minor numerical differences; however, the pattern remained the same. Despite the stabilization of depressive symptom rates, the gender gap persists, showing proportional improvement for both genders without closing the disparity.

The gender disparity observed in the SDQ total difficulties score warrants careful interpretation, as this score encompasses both internalizing and externalizing domains. Our analysis of subscales (Table S5) indicated that the elevated scores among females were primarily located in the emotional symptoms subscale, with no significant female predominance in conduct problems or hyperactivity/inattention. This pattern is consistent with normative SDQ data showing that emotional symptoms are more prevalent among female adolescents (Marzocchi et al., 2004). Indeed, the latter was slightly more prevalent among male adolescents, consistent with the externalizing pattern frequently observed in boys (Goodman, 2001). This finding suggests that the minor gender difference in the overall score conceals a substantial internalizing-specific disparity, which is somewhat offset by the absence of female predominance in externalizing issues. It also elucidates why the gender effect on the SDQ total score was most notably diminished following multivariable adjustment, as the behavioral and psychosocial covariates considered are more closely associated with internalizing rather than externalizing difficulties.

### 4.2. Gender Differences in Behavioral and Psychosocial Correlates

Physical activity showed the second largest gender difference among all behavioral variables. Male adolescents were substantially more likely than female adolescents to report at least three active days per week (75.6% vs. 59.8%; Cramér’s V = 0.169). This gap is consistent with the European HBSC survey findings, which consistently document lower physical activity levels in female adolescents, with widening disparities during mid-adolescence (Inchley et al., 2020; Ruiz-Ranz & Asín-Izquierdo, 2025). The association between gender and physical activity is relevant because physical inactivity may constitute a mechanistic pathway through which gender indirectly influences mental health, a possibility supported by the significant association between physical activity and depressive symptoms in the multivariable models.

Female adolescents reported sleep-onset difficulties more often (45.9% vs. 38.2%), aligning with evidence linking sleep disturbance to mental health issues in females (Goldstone et al., 2020; Lemke et al., 2025). Notably, late bedtime was similar across genders, indicating that differences in sleep quality stem from factors such as sleep onset difficulties or rumination. School stress was slightly higher among females (*p* = 0.004), supporting previous findings (Högberg et al., 2020), which found that school stress contributes to gender gaps in mental health in Sweden. Health literacy was similar between the genders (*p* = 0.934), matching the European HBSC data showing no gender advantage (Paakkari et al., 2020).

PIU (GPIUS-2) was similar across genders, but social media use (BSMAS) was slightly higher among females. This aligns with findings that gender differences in digital media are domain-specific: girls engage more in social networking, and boys in gaming (Cai et al., 2023). Females reported higher perceived social support from friends and significant others, reflecting gender differences in social orientation during adolescence (Rose & Rudolph, 2006). No gender difference in family support suggests that parental relationships do not affect mental health differently in males and females.

### 4.3. Multivariable Associations Between Correlates and Mental Health Outcomes

School stress showed the strongest association with all three mental health outcomes, confirming evidence from the COP-S series (Barbieri et al., 2024, 2026b) and European research that academic burden is a key risk factor for adolescent psychopathology (Hill et al., 2025; Högberg et al., 2020). School stress affects depressive symptoms, anxiety symptoms, and behavioral difficulties, making it a transdiagnostic risk factor. These findings suggest that reducing the academic burden may benefit mental health across multiple domains.

PIU showed the second strongest association overall, with the largest association observed for SDQ total difficulties, surpassing its association with depressive symptom and generalized anxiety symptom scores. This aligns with meta-analytic evidence linking PIU to conduct problems, hyperactivity, and peer difficulties (Wartberg et al., 2017). The strong SDQ association may reflect shared mechanisms related to impulse regulation, attentional difficulties, and peer conflict in both externalizing psychopathology and PIU (Cai et al., 2023). Notably, GPIUS-2 and BSMAS were collinear and could not be included in the same model; GPIUS-2 was retained as the primary predictor, given its broader conceptual scope. GPIUS-2 remained associated with all outcomes after adjustment for social media use, sleep, school stress, and social support, indicating that its association with mental health is not fully attributable to co-occurring factors. The strong associations between PIU and mental health symptoms require caution due to potential overlap between predictor and outcomes. The GPIUS-2 defines problematic Internet use partly through distress, impairment, and self-regulation issues, also reflected in depression, anxiety, and behavioral screening tools. Mood regulation, preoccupation, and negative consequences may overlap with symptom outcomes due to construct overlap rather than prediction. Additionally, using the same self-report instrument for both predictors and outcomes may inflate associations. These considerations do not invalidate PIU findings, consistent with evidence linking Internet use to adolescent psychopathology, but suggest the association’s magnitude should be seen as an upper-bound estimate. Future research using independent Internet behavior assessments, like screen-time logs or parent reports, could help clarify genuine prediction from construct overlap.

Sleep-onset difficulties were linked to all outcomes after adjustment, consistent with evidence that sleep issues predict adolescent psychopathology (Scott et al., 2021), and COP-S findings showing sleep as a consistent correlate (Barbieri et al., 2026b). Perceived social support had a protective effect and was strongest for SDQ behavioral difficulties. This supports the evidence that social connectedness buffers stress and aids coping (Scardera et al., 2020), and COP-S analyses identify low MSPSS as a predictor of mental health problems (Barbieri et al., 2026b).

The predictors showed outcome-specific associations. Late bedtimes were linked to depressive symptoms, not generalized anxiety symptom or SDQ scores, aligning with evidence that circadian phase delay affects depressive mood more than anxiety (Alvaro et al., 2013). Physical activity is inversely related to depressive symptoms, matching meta-analytic evidence of exercise’s stronger impact on depressive symptoms than anxiety symptoms in adolescents (Biddle & Asare, 2011). Health literacy was inversely associated with SDQ difficulties, suggesting the relevance of behavioral self-regulation over affective symptoms. Family affluence was not significantly linked to any outcome, echoing Barbieri et al.’s (2026b) findings of no association between parental education, material affluence, and self-reported mental health in this group of adolescents.

### 4.4. Attenuation of Gender Effects After Multivariable Adjustment

A distinctive feature of the present analysis is the joint, simultaneous-entry modelling of behavioral and psychosocial correlates across all three mental health domains, combined with the direct quantification of the proportion of the gender association that is shared with the measured correlates. The present study draws on the same 2025 wave of the COP-S survey as (Barbieri et al., 2026b), and the descriptive prevalence estimates are therefore consistent across the two analyses. The earlier publication examined within-gender predictors of mental health using gender-stratified logistic regression on dichotomized outcomes in a restricted sample. The present study shifts the focus to the gender gap itself, retains the continuous score range in a broader analytic sample, and provides the first formal comparison of differential attenuation across outcomes within this cohort.

The pattern of attenuation differed markedly across outcomes. For total difficulties, adjustment for the measured behavioral and psychosocial factors reduced the gender coefficient by 58.5%, such that the gender difference was no longer statistically detectable; physical activity, problematic Internet use, and sleep-onset difficulties, which differed by gender and were associated with the outcome, were the variables most likely to underlie this attenuation. This pattern is consistent with the hypothesis that interventions addressing these correlates might help to narrow the gender gap in emotional and behavioral difficulties, although this hypothesis would need to be tested in longitudinal or intervention studies. For depressive symptoms, the 37.1% attenuation indicates that approximately two-fifths of the unadjusted female excess was statistically attenuated by the measured factors, with the remainder consistent with the operation of unmeasured sex- or gender-specific mechanisms reported in cross-national studies (Michl et al., 2013; Rood et al., 2009). For generalized anxiety symptoms, only 25.3% of the unadjusted gender association was attenuated, indicating that the measured behavioral and psychosocial factors accounted for a comparatively small share of the female excess; the substantive interpretation of this residual is discussed in the following paragraph.

The limited attenuation of the gender effect on anxiety symptoms suggests mechanisms beyond behavioral and psychosocial factors. Biologically, pubertal transition involves ovarian hormone fluctuations and stress reactivity changes, coinciding with increased female internalizing symptoms from mid-adolescence; pubertal status was not assessed here (Hyde & Mezulis, 2020; Pfeifer & Allen, 2021). Socioculturally, female adolescents face appearance-related pressures and self-objectification, linked to appearance anxiety symptoms and depressive symptoms (Fredrickson & Roberts, 1997; Grabe et al., 2007). Cognitive-emotional styles also matter: female adolescents are prone to rumination and worry, which have been shown to account for part of the gender differences in depressive and anxiety symptoms, with worry being a particularly relevant factor for anxiety (Espinosa García et al., 2022; Johnson & Whisman, 2013). The SCARED-GAD items highlight worry about social evaluation and future events, suggesting cognitive vulnerability contributes to the residual gender effect on anxiety symptoms. These factors imply the residual female excess in anxiety symptoms reflects unmeasured biological, sociocultural, and cognitive influences, suggesting anxiety symptoms-specific preventive approaches should address appearance pressures, worry, and rumination, besides modifiable exposures identified here.

Overall, the findings align with a model in which gender differences in adolescent mental health are associated with behavioral and psychosocial factors that differ between genders and that are in principle modifiable, while residual gender-specific variation remains after their adjustment. The behavioral and psychosocial correlates identified here—school stress, sleep, problematic Internet use, social support, and physical activity—describe a coherent profile that may inform future prevention research, while recognizing that the cross-sectional design cannot establish their suitability as intervention targets.

### 4.5. Strengths and Limitations

This study had several strengths. The large regional sample closely matched the gender and age distribution of the South Tyrolean adolescent population. The use of three validated, internationally standardized screening instruments provides comprehensive coverage of adolescent mental health issues. The simultaneous entry of multiple behavioral and psychosocial factors in a single analytical framework enabled the assessment of relative predictor contributions of multiple correlates and the direct quantification of gender effect attenuation. The inclusion of both continuous and dichotomized outcome analyses provides complementary perspectives on the clinical burden and severity distribution. The present findings extend the prior COP-S 2025 dataset analyses. Barbieri et al. (2026b) identified within-gender predictors of mental health issues using gender-stratified logistic regression, finding school stress, PIU, sleep, and physical activity significant in both genders, with single parenthood and health literacy significant for females, and low social support for males. This study shifts the focus to the factors associated with the gender gap, using continuous outcomes and simultaneous entry regression for precise estimation of these associations and quantification of gender effect attenuation quantification, which have not been previously examined.

This study has several limitations. The cross-sectional design precludes causal inference. In the present analyses, gender and the behavioral and psychosocial variables are reported as correlates or statistical predictors of the mental health outcomes, not as causal risk factors; temporal ordering and confounding by unmeasured variables cannot be addressed within this design.

All predictors and outcomes were assessed by adolescent self-report obtained from a single online questionnaire administered at one time point. The design exposes all reported associations to common-method variance, whereby shared response tendencies and momentary states at the time of survey completion may inflate associations between predictors and outcomes to an unknown degree. Validated multi-item instruments mitigate this concern but do not eliminate it. Studies combining self-report with parent or teacher report, objective behavioral measures, or longitudinal designs that temporally separate predictor and outcome assessment would help to disentangle substantive associations from method-related variance.

The regression analyses were based on fewer participants than the descriptive base sample because of item-specific missingness in the behavioral and mental health modules. Each model retained only about 60% of the base sample (*n* = 1448–1500 of 2428, depending on the outcome), and the fully adjusted models were estimated on the subset of adolescents with complete data on all covariates. This reduction constitutes a central limitation of the study. To assess whether it introduced bias, analyzed and excluded participants were compared on all regression variables (Table S3). The groups did not differ significantly on any behavioral, psychosocial, or mental health variable; only age differed significantly, with a negligible effect size. This indicates that listwise deletion did not introduce substantial selection on the measured constructs, although the reduced statistical power and the possibility of residual unmeasured selection remain limitations.

The response rate of 23% may have introduced selection bias. School-mediated email recruitment may have systematically underrepresented families with limited digital access, migration backgrounds, or lower socioeconomic position. Comparison of the analytic sample (*n* = 2428) with regional reference data from the Provincial Statistics Institute (ASTAT) on the resident adolescent population of South Tyrol aged 11–19 years (*n* = 51,846 as of 31 December 2024) indicated close correspondence on gender distribution (48.6% vs. 48.0% female) and a modest overrepresentation of younger adolescents (48.1% vs. 44.0% aged 11–14 years; Table S2). Findings should therefore be interpreted as broadly reflective of, rather than strictly representative of, the regional adolescent population.

Conducted in a high-income, trilingual European region, which limits generalizability to setting with different educational, cultural, and economic contexts. Fifth, several relevant variables were not available, including clinical and treatment history; their absence may account for part of the gender differences that remained unexplained after adjustment.

Pubertal status was not assessed, a notable omission since pubertal timing and tempo are key developmental contributors to the female preponderance in internalizing symptoms during adolescence, operating partly independently of the factors examined. The unmeasured contribution of pubertal development may explain the residual gender effect on anxiety symptoms after adjustment. Without this variable, models cannot distinguish gender effects from social and behavioral factors versus pubertal maturation, affecting interpretations of the gender gap in internalizing symptoms.

The positive skewness of the PHQ-2 residuals (skewness 1.03) may reduce the precision of the depressive symptom model estimate, but the large sample size ensured asymptotic validity of the results.

The BSMAS and GPIUS-2 could not be entered simultaneously because of collinearity (rs 0.72); digital media findings therefore reflect PIU, and separate social media addiction examination was not possible.

Gender was treated as a binary variable, leading to the exclusion of the two adolescents who identified as gender-diverse due to the group’s insufficient size for separate analysis. Although this decision was statistically necessary, it fails to encompass the entire range of adolescent gender identities and restricts the relevance of the findings for gender-diverse youth, whose mental health needs might differ from those addressed here. Future research with larger samples should strive to include gender-diverse adolescents as a separate category rather than omitting them.

School stress, one of the most consistent correlates in this study, was measured with a single self-report item. Although single-item stress measures are widely used in large-scale adolescent health surveys and the item showed the expected associations, a single item cannot capture the multidimensional nature of academic stress, including its distinct sources such as performance pressure, workload, and social-evaluative concerns, and does not permit the estimation of internal consistency. The strength of the association between school stress and the mental health outcomes should therefore be interpreted with this measurement constraint in mind, and future studies would benefit from multi-item or validated academic stress scales.

## 5. Conclusions

Gender differences in adolescent mental health are consistent, with female adolescents scoring higher than male adolescents in the depressive symptom, anxiety symptoms, and emotional/behavioral domains. After comprehensive adjustment for behavioral and psychosocial factors, the gender difference remained statistically significant for anxiety and depressive symptom scores but was no longer significant for emotional and behavioral scores after adjustment for the measured covariates. The degree of attenuation after adjustment for modifiable exposures ranged from 25.3% for anxiety to 37.1% for depressive symptoms and 58.5% for emotional/behavioral difficulties, indicating that the gender difference in anxiety was least attenuated by the measured factors and warrants anxiety-specific preventive strategies targeting mechanisms beyond the behavioral correlates examined here. School stress and problematic Internet use were the factors most strongly and consistently associated with outcomes across the three mental health domains. Together with sleep-onset difficulties, physical activity, and perceived social support, these emerged as candidate factors for future prevention research and constitute plausible focal points for interventions to be evaluated in studies with a design that can support causal inference. Continued population-based monitoring through the COP-S survey series provides an essential foundation for evidence-based adolescent mental health policies in bilingual high-income regions and comparable settings.

## Figures and Tables

**Table 1:** Sociodemographic, behavioral, and mental health characteristics of the study sample (*n* = 2428), stratified by gender, with effect sizes.

Variable	Males (*n* = 1247)	Females (*n* = 1181)	Total (*n* = 2428)	*p*-Value	Effect Size
Sociodemographic characteristics					
Age, years—Median (min–max)	15 (11–19)	15 (11–19)	15 (11–19)	0.742 ^†^	r = 0.007
Mean ± SD	14.71 ± 2.33	14.74 ± 2.35	14.73 ± 2.34		
School type, *n* (%)				<0.001 *	V = 0.085
Middle school	519 (41.6)	468 (39.6)	987 (40.7)		
High school	591 (47.4)	629 (53.3)	1220 (50.2)		
Vocational school	122 (9.8)	68 (5.8)	190 (7.8)		
Other	15 (1.2)	16 (1.4)	31 (1.3)		
School language, *n* (%)				0.402 *	V = 0.027
German	1073 (86.7)	1005 (85.3)	2078 (86.0)		
Italian	137 (11.1)	138 (11.7)	275 (11.4)		
Ladin	27 (2.2)	35 (3.0)	62 (2.6)		
Family language, *n* (%)				0.312 *	V = 0.038
German	1016 (81.8)	942 (80.2)	1958 (81.0)		
Italian	186 (15.0)	178 (15.1)	364 (15.1)		
Ladin	25 (2.0)	36 (3.1)	61 (2.5)		
Other	15 (1.2)	19 (1.6)	34 (1.4)		
Migration background, *n* (%)	73 (6.0)	69 (5.9)	142 (5.9)	0.974 *	V = 0.001
Single-parent household, *n* (%)	144 (11.6)	159 (13.5)	303 (12.5)	0.150 *	V = 0.029
Parental education (CASMIN), *n* (%)				0.157 *	V = 0.039
Low	225 (18.2)	215 (18.4)	440 (18.3)		
Medium	493 (40.0)	507 (43.4)	1000 (41.6)		
High	516 (41.8)	446 (38.2)	962 (40.0)		
Urban residence, *n* (%)	371 (29.8)	336 (28.5)	707 (29.1)	0.481 *	V = 0.014
Family affluence (FAS-III), *n* (%)				0.004 *	V = 0.068
Low	178 (14.3)	218 (18.7)	396 (16.5)		
Medium	738 (59.4)	623 (53.5)	1361 (56.6)		
High	326 (26.2)	323 (27.7)	649 (27.0)		
Median (min–max)	9 (3–13)	9 (3–13)	9 (3–13)	0.172 ^†^	r = 0.028
Mean ± SD	9.36 ± 1.79	9.27 ± 1.93	9.31 ± 1.86		
Behavioral and lifestyle indicators					
Sleep difficulties ≥ 1×/week, *n* (%)	289 (38.2)	351 (45.9)	640 (42.1)	0.002 *	V = 0.079
Late bedtime on school days (>23:00), *n* (%)	103 (13.6)	89 (11.6)	192 (12.6)	0.247 *	V = 0.030
Physical activity ≥ 3×/week, *n* (%)	608 (75.6)	478 (59.8)	1086 (67.7)	<0.001 *	V = 0.169
Perceived global-crises burden, *n* (%)	300 (39.3)	344 (44.8)	644 (42.0)	0.028 *	V = 0.056
Problematic internet use (GPIUS-2 elevated), *n* (%)	190 (25.8)	218 (29.7)	408 (27.8)	0.093 *	V = 0.044
Total score, Median (min–max)	36 (15–120)	38 (15–110)	37 (15–120)	0.258 ^†^	r = 0.030
Mean ± SD	39.46 ± 19.22	40.74 ± 19.83	40.10 ± 19.53		
Perceived social support (MSPSS) category, *n* (%)				0.943 *	V = 0.009
Low	54 (6.8)	55 (7.0)	109 (6.9)		
Moderate	92 (11.5)	87 (11.0)	179 (11.3)		
High	651 (81.7)	646 (82.0)	1297 (81.8)		
Total score, Median (min–max)	6.17 (1–7)	6.25 (1–7)	6.17 (1–7)	0.043 ^†^	r = 0.051
Mean ± SD	5.76 ± 1.45	5.83 ± 1.44	5.79 ± 1.44		
Health literacy (HLSAC) category, *n* (%)				0.600 *	V = 0.026
Low	73 (10.0)	84 (11.5)	157 (10.8)		
Medium	494 (68.0)	480 (65.9)	974 (66.9)		
High	160 (22.0)	164 (22.5)	324 (22.3)		
Total score, Median (min–max)	32 (10–40)	32 (10–40)	32 (10–40)	0.883 ^†^	r = 0.004
Mean ± SD	31.59 ± 5.03	31.56 ± 5.09	31.58 ± 5.06		
Mental health screening results					
PHQ-2 elevated (≥3), *n* (%)	67 (9.0)	104 (13.8)	171 (11.4)	0.003 *	V = 0.076
PHQ-2 total score, Median (min–max)	1 (0–6)	1 (0–6)	1 (0–6)	<0.001 ^†^	r = 0.134
Mean ± SD	0.90 ± 1.15	1.28 ± 1.44	1.09 ± 1.32		
SCARED GAD-9 elevated (≥ 9), *n* (%)	144 (19.5)	278 (37.6)	422 (28.5)	<0.001 *	V = 0.200
SCARED total score, Median (min–max)	4 (0–18)	6 (0–18)	5 (0–18)	<0.001 ^†^	r = 0.236
Mean ± SD	4.94 ± 4.19	7.21 ± 4.95	6.08 ± 4.72		
SDQ elevated (borderline/abnormal), *n* (%)	82 (11.4)	123 (16.9)	205 (14.2)	0.003 *	V = 0.079
SDQ total score, Median (min–max)	8 (0–30)	8 (0–30)	8 (0–30)	0.002 ^†^	r = 0.081
Mean ± SD	8.51 ± 5.32	9.58 ± 5.99	9.05 ± 5.69		

Values are presented as *n* (%) for categorical variables and as median (minimum–maximum) or mean ± standard deviation (SD) for continuous variables. The denominators varied because of item-specific missingness; *n* reflects the number of cases with valid data for each variable. Valid data were available for 2402–2428 adolescents (98.9–100%) for sociodemographic characteristics, 1455–1604 (59.9–66.1%) for behavioral and lifestyle indicators, and 1448–1500 (59.6–61.8%) for mental health screening. Gender comparisons: * Pearson χ^2^ test for categorical variables, with Cramér’s V (V) as effect size; ^†^ Mann–Whitney U test for continuous and ordinal variables, with rank-biserial correlation coefficient r = |Z|/√N as effect size: V or r ≈ 0.10 indicates a small, ≈0.30 a medium, and ≈0.50 a large effect (Cohen, 1988). Abbreviations: CASMIN, Comparative Analysis of Social Mobility in Industrial Nations; FAS-III, Family Affluence Scale III; GPIUS-2, Generalized Problematic Internet Use Scale 2; HLSAC, Health Literacy for School-Aged Children; MSPSS, Multidimensional Scale of Perceived Social Support; PHQ-2, Patient Health Questionnaire-2; SCARED, Screen for Child Anxiety-Related Emotional Disorders; SDQ, Strengths and Difficulties Questionnaire.

**Table 2:** Multivariable linear regression models for depressive symptoms (PHQ-2), generalized anxiety symptoms (SCARED-GAD), and emotional/behavioral (SDQ) scores.

Variable	PHQ-2 Depressive Symptoms (*n* = 1329)				SCARED-GAD Generalized Anxiety Symptoms (*n* = 1311)				SDQ Total Difficulties (*n* = 1275)			
	B	95% CI	β	*p*	B	95% CI	β	*p*	B	95% CI	β	*p*
Gender (female)	0.23	[0.12, 0.35]	0.09	<0.001	1.70	[1.27, 2.12]	0.18	<0.001	0.44	[−0.05, 0.92]	0.04	0.078
Age	0.06	[0.03, 0.08]	0.09	<0.001	0.07	[−0.03, 0.17]	0.03	0.174	−0.15	[−0.26, −0.03]	−0.06	0.011
Physical activity	−0.06	[−0.09, −0.02]	−0.08	<0.001	−0.13	[−0.24, −0.01]	−0.05	0.036	−0.10	[−0.23, 0.04]	−0.03	0.150
School stress ^1^	0.35	[0.28, 0.42]	0.23	<0.001	1.53	[1.27, 1.79]	0.28	<0.001	1.56	[1.26, 1.85]	0.24	<0.001
HLSAC	0.00	[−0.01, 0.01]	0.01	0.763	−0.02	[−0.06, 0.03]	−0.02	0.429	−0.12	[−0.16, −0.07]	−0.10	<0.001
Sleep-onset difficulties	0.48	[0.37, 0.61]	0.18	<0.001	1.96	[1.52, 2.40]	0.20	<0.001	1.95	[1.45, 2.45]	0.17	<0.001
Late bedtime	0.29	[0.11, 0.47]	0.07	0.002	0.30	[−0.35, 0.96]	0.02	0.365	0.48	[−0.27, 1.23]	0.03	0.213
PIU (GPIUS-2)	0.02	[0.01, 0.02]	0.26	<0.001	0.06	[0.05, 0.07]	0.24	<0.001	0.11	[0.10, 0.12]	0.38	<0.001
MSPSS	−0.14	[−0.19, −0.10]	−0.15	<0.001	−0.25	[−0.40, −0.09]	−0.07	0.002	−0.67	[−0.85, −0.50]	−0.16	<0.001
FAS-III	0.02	[−0.01, 0.05]	0.02	0.271	0.06	[−0.05, 0.18]	0.03	0.265	−0.04	[−0.17, 0.09]	−0.01	0.542
R^2^ (adjusted R^2^)	0.36 (0.36)				0.35 (0.35)				0.43 (0.42)			
F (df1, df2)	74.66 (10, 1318)				71.31 (10, 1300)				94.53 (10, 1264)			
Model *p*-value	<0.001				<0.001				<0.001			
VIF range	1.02–1.18				1.02–1.18				1.02–1.18			

B, unstandardized regression coefficient; 95% CI, 95% confidence interval for B; β, standardized regression coefficient. All models used simultaneous entry (Enter method) with listwise deletion of missing values. Gender was coded 0 = male, 1 = female. PHQ-2 range: 0–6; SCARED-GAD range: 0–18; SDQ total difficulties range: 0–40. ^1^ School stress was entered as a metric covariate on the original four-point response scale (1 = not at all, 4 = very strongly). Late bedtime denotes a reported bedtime on school days after 23:00. Abbreviations: FAS-III, Family Affluence Scale III; GPIUS-2, Generalized Problematic Internet Use Scale 2; HLSAC, Health Literacy for School-Aged Children; MSPSS, Multidimensional Scale of Perceived Social Support; PIU, problematic Internet use; VIF, variance inflation factor.

**Table 3:** Unadjusted and adjusted associations between gender and mental health outcomes, with attenuation of the gender coefficient after adjustment for behavioral and psychosocial factors.

Mental Health Outcome	Unadjusted Model		Adjusted Model			Attenuation of β
	B (SE)	β [95% CI]; *p*-Value	B (SE)	β; *p*-Value	95% CI for B	
Depressive symptoms (PHQ-2)	0.377 (0.067)	0.143 [0.244, 0.509]; <0.001	0.234 (0.059)	0.090; <0.001	[0.117, 0.350]	37.1%
Generalized anxiety symptoms (SCARED-GAD)	2.273 (0.239)	0.241 [1.805, 2.741]; <0.001	1.695 (0.217)	0.180; <0.001	[1.268, 2.121]	25.3%
Total difficulties (SDQ)	1.066 (0.298)	0.094 [0.482, 1.650]; <0.001	0.436 (0.247)	0.039; 0.078	[−0.049, 0.921]	58.5%

B, unstandardized regression coefficient; SE, standard error; β, standardized regression coefficient; CI, confidence interval. Unadjusted models regressed each outcome on gender alone (*n* = 1500, 1479, and 1448 for PHQ-2, SCARED-GAD, and SDQ, respectively). Adjusted models additionally included age, physical activity, school stress, health literacy (HLSAC), sleep-onset difficulties, late bedtime, problematic Internet use (GPIUS-2), perceived social support (MSPSS total), and family affluence (FAS III), and were estimated by listwise deletion (*n* = 1329, 1311, and 1275; adjusted R^2^ = 0.36, 0.35, and 0.42). Gender was coded 0 = male, 1 = female. Attenuation of β was computed as ((β unadjusted − β adjusted)/β unadjusted) × 100, following established change-in-estimate conventions (Greenland, 1989; MacKinnon, 2012); it is a descriptive measure of the degree to which the gender coefficient is reduced after covariate adjustment and does not imply mediation or a causal pathway.

## Data Availability

The data presented in this study are available from the corresponding author upon reasonable request.

## Supplementary Materials

The following supporting information can be downloaded at https://www.mdpi.com/article/10.3390/bs16050812/s1, Table S1: Spearman rank-order correlations among study variables (*n* = 2428, pairwise deletion); Table S2: Comparison of the analytic sample (*n* = 2068) with the resident adolescent population of South Tyrol (11–19 years) on key sociodemographic characteristics; Table S3: Sensitivity analysis: comparison of analyzed and excluded participants on all regression variables; Table S4: Sensitivity analysis: comparison of the gender association across ordinary least squares (OLS) and Poisson generalized linear models for the three mental health outcomes; Table S5: Gender differences in the Strengths and Difficulties Questionnaire (SDQ) subscales and total difficulties score.

## Abbreviations

The following abbreviations are used in this manuscript:

AI	Artificial intelligence
BSMAS	Bergen Social Media Addiction Scale
CASMIN	Comparative Analysis of Social Mobility in Industrial Nations
COP-S	Corona and Psyche South Tyrol
FAS III	Family Affluence Scale III
GAD-9	Generalized Anxiety Disorder subscale
GPIUS-2	Generalized Problematic Internet Use Scale 2
HBSC	Health Behavior in School-aged Children
HLSAC	Health Literacy for School-Aged Children
IQR	Interquartile range
IRR	Incidence rate ratio
MSPSS	Multidimensional Scale of Perceived Social Support
OLS	Ordinary least squares
PHQ-2	Patient Health Questionnaire-2
PIU	Problematic internet use
SCARED	Screen for Child Anxiety Related Emotional Disorders
SDQ	Strengths and Difficulties Questionnaire
VIF	Variance inflation factor
