# Supplementary material for: Behavioral and Psychosocial Correlates of Gender Differences in Adolescent Mental Health: A Regional Cross-Sectional Study in Northern Italy
Source: Behav Sci (Basel). 2026 May 19;16(5):812. doi: 10.3390/bs16050812 (PMC13203345; doi:10.3390/bs16050812)
Supplement: Supplementary file 1 [file behavsci-16-00812-s001.zip › behavsci-4310071-supplementary.pdf]

Supplementary Materials

Table S1. Spearman rank-order correlations among study variables (N = 2,428, pairwise deletion).

Variable	1	2	3	4	5	6	7	8	9	10	11	12	13	14	15	16
1. PHQ-2	1.000															
2. SCARED	0.551**	1.000														
3. SDQ	0.571**	0.635**	1.000													
4. PhysAct	-0.196**	-0.198**	-0.162**	1.000												
5. SchStress	0.384**	0.409**	0.385**	-0.130**	1.000											
6. HlthLit	-0.155**	-0.171**	-0.289**	0.122**	-0.153**	1.000										
7. PoorSlp	0.336**	0.348**	0.332**	-0.104**	0.233**	-0.101**	1.000									
8. LateBed	0.198**	0.143**	0.144**	-0.101**	0.185**	-0.011	0.166**	1.000								
9. GPIUS-2	0.421**	0.403**	0.511**	-0.175**	0.227**	-0.242**	0.221**	0.152**	1.000							
10. BSMAS	0.381**	0.394**	0.431**	-0.152**	0.237**	-0.184**	0.200**	0.147**	0.720**	1.000						
11. MSPSS-F	-0.310**	-0.235**	-0.342**	0.078**	-0.166**	0.213**	-0.170**	-0.123**	-0.314**	-0.292**	1.000					
12. MSPSS-Fr	-0.245**	-0.219**	-0.317**	0.071**	-0.165**	0.183**	-0.129**	-0.015	-0.176**	-0.146**	0.579**	1.000				
13. MSPSS-O	-0.222**	-0.153**	-0.250**	0.064*	-0.107**	0.221**	-0.109**	-0.069**	-0.240**	-0.195**	0.741**	0.684**	1.000			
14. FAS	0.019	0.022	-0.051	0.073**	-0.021	0.084**	0.021	0.022	0.012	0.014	0.026	0.024	0.028	1.000		
15. ParEduc	-0.007	0.004	-0.020	0.098**	-0.016	0.124**	0.014	0.019	-0.022	-0.076**	0.049	0.030	0.027	0.257**	1.000	
16. Age	0.216**	0.132**	0.056*	-0.168**	0.167**	0.065*	0.057*	0.306**	0.145**	0.208**	-0.111**	0.006	-0.077**	0.032	-0.025	1.000

Values are Spearman rank-order correlation coefficients (r_s). ** $p < 0.01$, * $p < 0.05$ (two-tailed). Cells highlighted in yellow indicate $r_s \geq 0.70$ (collinearity threshold). The only predictor pair exceeding this threshold was GPIUS-2 × BSMAS ($r_s = 0.720$); accordingly, BSMAS was excluded from multivariable models and GPIUS-2 was retained as the primary digital media predictor. The MSPSS Family × Significant Others subscale correlation ($r_s = 0.741$) motivated the use of the MSPSS total score in place of individual subscales in the regression models. Variable abbreviations: PHQ-2, Patient Health Questionnaire-2 (depressive score); SCARED, Screen for Child Anxiety Related Emotional Disorders — GAD-9 subscale (anxiety score); SDQ, Strengths and Difficulties Questionnaire total difficulties score; PhysAct, physical activity (days/week ≥ 60 min); SchStress, self-reported school stress (single item); HlthLit, Health Literacy for School-Aged Children (HLSAC) total score; PoorSlp, poor sleep quality (difficulty falling asleep ≥ 1×/week); LateBed, habitual school-day bedtime after 23:00; GPIUS-2, Generalized Problematic Internet Use Scale 2 total score; BSMAS, Bergen Social Media Addiction Scale total score; MSPSS-F, MSPSS Family subscale; MSPSS-Fr, MSPSS Friends subscale; MSPSS-O, MSPSS Significant Others subscale; FAS, Family Affluence Scale III total score; ParEduc, parental education (CASMIN, ordinal); Age, adolescent age in years.

Table S2. Comparison of the analytic sample (n = 2068) with the resident adolescent population of South Tyrol (11–19 years) on key sociodemographic characteristics.

Characteristic	COP-S analytic sample (n = 2428)	South Tyrolean reference population (11–19 years)	Difference (percentage points)
Gender			
Male, n (%)	1247 (51.4)	26 963 (52.0)	−0.6
Female, n (%)	1181 (48.6)	24 883 (48.0)	+0.6
Age group			
11–14 years, n (%)	1167 (48.1)	22 787 (44.0)	+4.1
15–19 years, n (%)	1261 (51.9)	29 059 (56.0)	−4.1
Sampling coverage			
Analytic sample as % of resident 11–19-year-olds	2428 / 51 846 = 4.7 %		

Reference population data are derived from the Provincial Statistics Institute (ASTAT) of the Autonomous Province of Bolzano–South Tyrol, resident population by single year of age and gender as of 31 December 2024. The COP-S 2025 survey was fielded between 17 March and 13 April 2025; the 31 December 2024 census represents the closest available reference time point. The analytic sample comprises adolescent self-report records aged 11–19 years attending lower secondary school or above (base sample for descriptive analyses). Abbreviations: ASTAT, Provincial Statistics Institute of South Tyrol; COP-S, Corona and Psyche South Tyrol survey. Source: ASTAT, Wohnbevölkerung nach Alter und Geschlecht, 31.12.2024. Available at: https://statastat.prov.bz.it/databrowser/#/de/dissemination_node/categories/ITH1,DISS_DEMO_SOCIAL_STAT,1.0/POP_MIGRATION/POPULATION/POP_OFFICIAL/ITH1,DF_POPMIG_POPRES_3,1.0 (accessed 14 May 2026).

Table S3. Sensitivity analysis: comparison of analyzed and excluded participants on all regression variables.

Variable	Analyzed [1]	Excluded (listwise)	Test	*p*	Effect size
Sociodemographic					
Age, median (years)	15.0	15.0	MWU	0.003	r = 0.06
Female, %	49.6	47.6	χ^2	0.304	V = 0.02
Family affluence (FAS III), median	9.0	9.0	MWU	.574	r = 0.01
Behavioral and psychosocial					
Physical activity, median	4.0	4.0	MWU	0.449	r = 0.02
School stress, median	2.0	2.0	MWU	0.115	r = 0.04
Health literacy (HLSAC), median	32.0	31.0	MWU	0.355	r = 0.02
PIU (GPIUS-2), median	37.0	36.0	MWU	0.981	r = 0.00
Social support (MSPSS), median	6.17	6.17	MWU	0.265	r = 0.03
Poor sleep quality, %	42.1	41.9	χ^2	0.956	V = 0.00

Variable	Analyzed [1]	Excluded (listwise)	Test	*p*	Effect size
		Mental health outcomes			
PHQ-2, median	1.0	1.0	MWU	0.576	r = 0.01
SCARED-GAD, median	5.0	5.0	MWU	0.358	r = 0.02
SDQ total difficulties, median	8.0	9.0	MWU	0.069	r = 0.05

[1] Participants in the base sample (n = 2428) were classified as analyzed (complete data on all regression variables; n = 1261) or excluded by listwise deletion (n = 1167). Continuous and ordinal variables were compared with the Mann–Whitney U test (MWU; effect size r = |Z|/√N); categorical variables with the Pearson χ^2 test (effect size Cramér's V). Group sizes for individual variables vary because of item-specific missingness among excluded participants. Abbreviations: FAS III, Family Affluence Scale III; GPIUS-2, Generalized Problematic Internet Use Scale 2; HLSAC, Health Literacy for School-Aged Children; MSPSS, Multidimensional Scale of Perceived Social Support; PHQ-2, Patient Health Questionnaire-2; PIU, problematic Internet use; SCARED-GAD, Screen for Child Anxiety-Related Emotional Disorders, generalized anxiety subscale; SDQ, Strengths and Difficulties Questionnaire.

Table S4. Sensitivity analysis: comparison of the gender association across ordinary least squares (OLS) and Poisson generalized linear models for the three mental health outcomes.

Outcome	OLS model (standardized)		Poisson GLM with robust SE		
	β	*p*-value	IRR	95% CI	*p*-value
Depressive symptoms (PHQ-2)	0.09	< 0.001	1.21	[1.09, 1.34]	< 0.001
Anxiety symptoms (SCARED-GAD)	0.18	< 0.001	1.32	[1.23, 1.43]	< 0.001
Total difficulties (SDQ)	0.04	0.078	1.04	[0.98, 1.10]	0.189

The table compares the gender association estimated by the main OLS regression models (standardized coefficient β) with that estimated by Poisson generalized linear models with a log link (incidence rate ratio, IRR = exp(B)). Gender was coded 0 = male, 1 = female; an IRR above 1 indicates a higher expected symptom score for female adolescents. All models were adjusted for the same set of covariates (age, physical activity, school stress, health literacy, poor sleep quality, late bedtime, problematic Internet use, perceived social support, and family affluence). Robust (Huber–White sandwich) standard errors were used for all Poisson models. The deviance/df ratio indicated no overdispersion for the PHQ-2 model (1.05) and moderate overdispersion for the SCARED-GAD (2.74) and SDQ (2.26) models; the robust standard errors provide valid inference under these conditions. Across all three outcomes, the OLS and Poisson models yielded the same inferential conclusion regarding the gender association, supporting the robustness of the main analyses to the skewness, bounded range, and floor effects of the outcome scales. Abbreviations: CI, confidence interval; GLM, generalized linear model; IRR, incidence rate ratio; OLS, ordinary least squares; SE, standard error.

Table S5. Gender differences in the Strengths and Difficulties Questionnaire (SDQ) subscales and total difficulties score.

SDQ subscale	Males, median (mean)	Females, median (mean)	*p*-value	Effect size (r)
Emotional symptoms	1.0 (1.87)	3.0 (3.05)	< 0.001	0.24
Conduct problems	1.0 (1.58)	1.0 (1.51)	0.165	0.04
Hyperactivity/inattention	3.0 (3.31)	3.0 (2.93)	< 0.001	0.09

SDQ subscale	Males, median (mean)	Females, median (mean)	*p*-value	Effect size (r)
Peer relationship problems	1.0 (1.81)	2.0 (2.03)	0.035	0.05
Prosocial behaviour	8.0 (8.03)	9.0 (8.55)	< 0.001	0.14
Total difficulties score	8.0 (8.51)	8.0 (9.58)	0.002	0.08

Self-reported SDQ subscales (child version) in the analytic sample. Group sizes ranged from 1492 to 1509 for the subscales and were 1448 for the total difficulties score, varying because of item-specific missingness. Each subscale ranges from 0 to 10; the total difficulties score is the sum of the emotional symptoms, conduct problems, hyperactivity/inattention, and peer relationship problems subscales (range 0–40) and does not include the prosocial behavior subscale. Higher prosocial scores indicate more favorable functioning. Groups were compared using the Mann–Whitney U test; the effect size $r = |Z|/\sqrt{N}$ is interpreted as negligible (< 0.10), small (0.10–0.29), moderate (0.30–0.49), or large (≥ 0.50). The female excess in the total difficulties score was driven almost entirely by the emotional symptoms subscale, which showed the largest gender difference (r = 0.24); the externalizing subscales showed no female excess, with hyperactivity/inattention slightly higher among male adolescents. Abbreviations: SDQ, Strengths and Difficulties Questionnaire.
